# An AI-Enabled Single-Cell Transcriptomic Analysis Pipeline for Gene Signature Discovery in Natural Killer Cells Linked to Remission Outcomes in Chronic Myeloid Leukemia

**DOI:** 10.3390/biology15070588

**Published:** 2026-04-06

**Authors:** Santoshi Borra, Da Yan, Robert S. Welner, Zongliang Yue

**Affiliations:** 1 Department of Computer Sciences, Luddy School of Informatics, Computing, and Engineering (SICE), Indiana University, Bloomington, IN 47408, USA; vborra@iu.edu (S.B.); yanda@iu.edu (D.Y.); 2 Division of Hematology and Oncology, Department of Medicine, O’Neal Comprehensive Cancer Center, University of Alabama at Birmingham, Birmingham, AL 35294, USA; rswelner@uabmc.edu; 3 Department of Health Outcomes Research and Policy, Harrison College of Pharmacy, Auburn University, Auburn, AL 36849, USA

**Keywords:** integrative bioinformatics, gene regulatory network (GRN) inference, machine learning-based gene panel discovery, chronic myeloid leukemia (CML), single-cell transcriptomics analysis

## Abstract

Background: A major technical challenge in single-cell transcriptomics is the absence of an integrative analytic pipeline that can simultaneously leverage gene regulatory network (GRN) architecture, AI-assisted gene panel discovery, and functional relevance analyses to generate coherent biological insights. Existing approaches often treat these components independently, focusing on clusters, marker genes, or predictive features without integrating them into a mechanistically grounded framework. Consequently, comprehensive screening that links regulatory association, gene signature screening, and functional interpretation within single-cell datasets remains limited, underscoring the need for an integrated strategy. Methods: We developed an integrative bioinformatics pipeline based on Gene regulatory network–AI–Functional Analysis (GAFA), combining latent-space integration, unsupervised clustering, diffusion pseudotime analysis, lineage-resolved generalized additive modeling, GRN inference, and machine learning-based gene panel discovery. This framework enables systematic mapping of cell-state structure, reconstruction of differentiation and effector trajectories, and identification of transcriptional and regulatory features strongly associated with clinical outcomes. As a case study, we applied the pipeline to NK cell transcriptomes from six CML patients (two early relapse, two late relapse, two durable treatment-free remission—TFR; 15 samples) collected at TKI discontinuation and 6–12 months after therapy cessation. Results: We reanalyzed publicly available scRNA-seq data from a previously published CML cohort to evaluate NK-cell transcriptional programs associated with treatment-free remission and relapse. We resolved six transcriptionally distinct NK cell states spanning CD56^bright^-like cytokine-responsive, early activated, terminally mature, cytotoxic, lymphoid trafficking, and HLA-DR^+^ immunoregulatory populations, each exhibiting outcome-specific compositional differences. Pseudotime analysis revealed two major NK cell lineages—a maturation trajectory and a cytotoxic effector trajectory. TFR samples displayed balanced occupancy of both lineages, whereas early relapse samples showed marked depletion of the maturation branch and preferential accumulation in cytotoxic end states. AI-guided feature selection and random forest modeling identified an 18-gene panel that distinguished NK cells from TFR and relapse samples in an exploratory manner. Among them, *CST7*, *FCER1G*, *GNLY*, *GZMA*, and *HLA-C* were conventional NK-associated genes, whereas *ACTB*, *CYBA*, *IFITM2*, *IFITM3*, *LYZ*, *MALAT1*, *MT2A*, *MYOM2*, *NFKBIA*, *PIM1*, *S100A8*, *S100B*, and *TSC22D3* were novel. The GRN inference further uncovered outcome-specific regulatory modules, with *RUNX3*, *EOMES*, *ELK4*, and *REL* regulons enriched in TFR, whereas *FOSL2* and *MAF* regulons were enriched in relapse, and their downstream targets linked to IFN-γ signaling, metabolic reprogramming, and immunoregulatory feedback circuits. Conclusions: This AI-enabled single-cell analysis demonstrates how NK cell state composition, differentiation trajectories, and regulatory network rewiring collectively shape TFR versus relapse following TKI discontinuation in CML. The integrative pipeline provides a modular framework that could be extended to additional datasets for data-driven biomarker discovery and mechanistic stratification, and highlights candidate transcriptional regulators and NK cell programs that may be leveraged to improve remission durability, pending validation in larger patient cohorts.

## Simple Summary

We developed an integrative analysis pipeline, Gene regulatory network–AI–Functional Analysis (GAFA) that advances single-cell data analysis, linking how immune cells change over time, which genes control those changes, and which genes distinguish patient outcomes in exploratory analysis. Most existing workflows run these steps separately (clustering, trajectories, regulatory networks, and machine learning), which makes it hard to explain why certain gene signatures matter. GAFA aims to address this gap by integrating them into one coherent framework, allowing us to discover clinical outcome-relevant NK cell regulatory programs and compact gene panels that may help predict whether CML patients will stay in treatment-free remission or relapse after stopping therapy.

## 1. Introduction

To identify predictive single-cell gene signatures and uncover the molecular mechanisms underlying distinct cellular phenotypes, integrative analytical strategies are required that move beyond purely data-driven approaches to explicitly incorporate systems biology and functional interpretation [1,2]. Comprehensive insight from single-cell transcriptomic data demands analytical frameworks that can unify regulatory architecture, predictive modeling, and functional relevance within a coherent and mechanistically grounded paradigm. The integrative Gene regulatory network–AI–Functional Analysis (GAFA) framework simultaneously leverages gene regulatory network (GRN) architecture [3,4,5,6], artificial intelligence-assisted gene panel discovery [7,8,9], and functional relevance analysis [10,11,12,13] to generate biologically interpretable and outcome-relevant insights. Despite substantial advances in single-cell RNA sequencing technologies and associated computational tools, most existing analytical workflows treat key components—such as dimensionality reduction and clustering, differential expression analysis, trajectory inference, GRN reconstruction, and machine learning-based feature selection—as sequential or independent steps rather than as interdependent representations of a unified biological system [14]. Consequently, latent-space embeddings, pseudotime trajectories, regulatory network inference, and predictive modeling are rarely reconciled in a principled manner that preserves regulatory causality inference, lineage structure, and functional coherence. This methodological fragmentation limits the ability to connect transcription factor-centric regulatory programs with clinically informative gene panels and their functional roles across dynamic cell-state transitions. Particularly, GRN inference is frequently performed post hoc on pre-defined clusters [15,16], trajectory analysis is decoupled from regulatory modeling [17,18], and machine learning approaches often prioritize predictive performance without incorporating regulatory or developmental constraints [19,20]. As a result, comprehensive screening strategies that integrate regulatory topology, lineage-resolved transcriptional dynamics, and AI-guided gene prioritization remain scarce. Addressing this gap requires AI-enabled integrative bioinformatics frameworks, such as GAFA, that explicitly couple latent-space representation, trajectory-aware modeling, GRN reconstruction, and supervised feature selection to enable mechanistically informed hypotheses and translational insights from single-cell datasets.

Integrative single-cell analytics are critical for studying complex diseases such as chronic myeloid leukemia (CML), providing a clinically compelling context in which biologically and translationally meaningful insights can be systematically derived. Driven by the BCR–ABL1 fusion tyrosine kinase arising from the Philadelphia chromosome translocation t(9;22)(q34;q11), CML is characterized by constitutive oncogenic signaling and unchecked expansion of myeloid progenitors [21]. The introduction of tyrosine kinase inhibitors (TKIs), beginning with imatinib, has transformed CML from a fatal malignancy into a chronic condition with long-term survival exceeding 90% [22,23]. As a result, treatment goals have shifted from disease control to therapy optimization, particularly minimizing cumulative toxicity, financial burden, and quality-of-life impairment associated with lifelong TKI exposure. TFR, the sustained maintenance of deep molecular response following TKI discontinuation, has therefore emerged as a central objective in contemporary CML management [23]. Although large clinical trials such as EURO-SKI [24] and DASFREE [25] demonstrate that approximately 40–60% of eligible patients can successfully maintain TFR, a substantial proportion experience molecular relapse and require treatment resumption [24,25]. The divergence of outcomes among clinically comparable patient samples highlights a critical unmet need to identify molecular and cellular determinants of durable remission, ideally in the form of gene signatures that capture underlying biological mechanisms rather than surface-level phenotypes.

Mounting evidence implicates immune surveillance, particularly natural killer (NK) cell-mediated control, as a key biological determinant of TFR success following TKI withdrawal [26]. Patients who sustain TFR consistently exhibit higher frequencies or absolute counts of mature, cytotoxic NK cell subsets at the time of discontinuation, whereas those who relapse often display functional impairment characterized by reduced degranulation capacity, diminished IFN-γ production, and increased expression of inhibitory or exhaustion-associated receptors [24,26]. These observations suggest that NK cell competence is critical for restraining residual leukemic cells once pharmacologic suppression is removed. However, existing studies, including those leveraging single-cell RNA sequencing, have largely focused on descriptive features such as population composition, marker gene expression, or global transcriptional shifts [27,28,29,30]. The regulatory programs, gene-regulatory network architectures, and lineage-resolved transcriptional dynamics that distinguish NK cells in durable TFR from those in early or late relapse remain poorly defined. Critically, no prior work has systematically integrated GRN structure, trajectory-aware modeling, and AI-guided gene selection to derive mechanistically interpretable and outcome-predictive NK cell gene signatures. This analytical gap directly motivates the application of the GAFA framework, which is uniquely positioned to integrate regulatory association, developmental dynamics, and functional relevance to identify novel NK cell gene panels with exploratory predictive capacity for distinct cells from TFR and relapse samples in the setting of TKI discontinuation.

To address these challenges, we developed an integrative bioinformatics pipeline using the GAFA framework for the single-cell analysis of 15 scRNA-seq samples from six CML patients collected before and after imatinib discontinuation, encompassing three distinct clinical trajectories: durable TFR, early relapse, and late relapse. By jointly leveraging latent-space representation learning, diffusion-based pseudotime reconstruction, and gene regulatory network inference, we delineated transcription factor-centric regulatory modules and inferred their functional roles in NK cell activation, differentiation, and exhaustion programs associated with divergent clinical outcomes. We further integrated network-aware modeling with supervised machine learning classifiers to identify predictive candidate NK cell gene signatures associated with outcome. This AI-enabled analytical strategy integrates cell-state dynamics with functional interpretation within a unified framework, facilitating the identification of regulatory patterns that may be less apparent in conventional single-cell workflows. To avoid purely performance-driven feature selection, supervised machine learning models were constrained by trajectory-informed gene dynamics and GRN-derived regulatory structure. Collectively, our findings provide mechanistic insight into NK cell-mediated immune surveillance following TKI discontinuation in CML and identify hypothesis-generating gene signatures that may inform immunologically guided strategies to improve the durability of TFR.

## 2. Materials and Methods

### 2.1. Data Source, Preprocessing, and Pipeline Design

**Figure 1:**
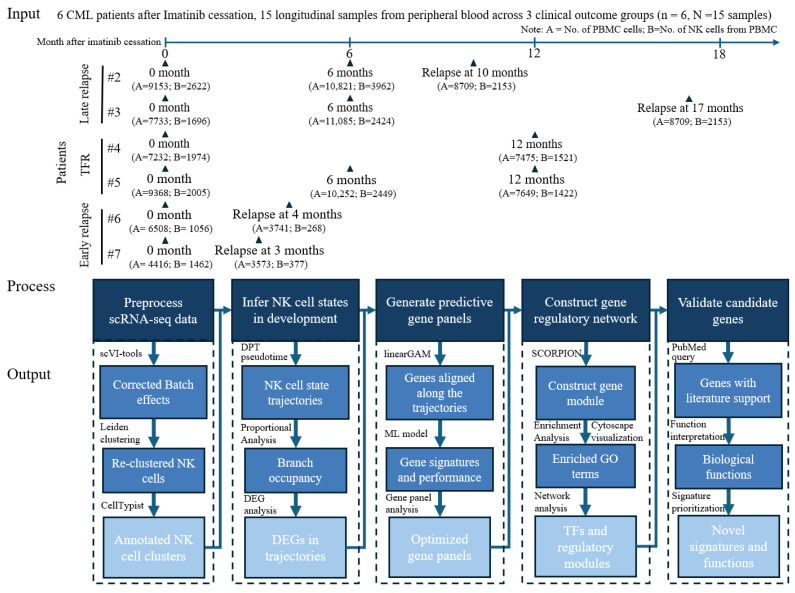
Overview of the scRNA-seq dataset and analytical workflow used in this study. Single-cell RNA sequencing was performed on natural killer (NK) cells collected from patients with chronic myeloid leukemia (CML). The dataset comprises 15 samples from six patients, collected across multiple time points, including baseline, 6 months, 12 months, and relapse. Clinical outcomes represented in the cohort include treatment-free remission (TFR), early relapse, and late relapse. The analytical workflow consists of five major steps: (1) preprocess scRNA-seq data, (2) infer NK cell states in development, (3) generate predictive gene panels, (4) construct gene regulatory networks, and (5) validate candidate gene modules. The resulting outputs include annotated NK cell clusters, trajectory-associated differentially expressed genes (DEGs), optimized predictive gene panels, transcription factors (TFs) and their regulatory modules, and novel gene signatures with enriched functions.

The scRNA-seq dataset analyzed in this study was originally generated from peripheral blood samples collected from six patients with CML at defined treatment checkpoints surrounding TKI discontinuation, capturing molecular remission, early relapse, and late relapse outcomes (Figure 1). These longitudinal samples were profiled using droplet-based single-cell RNA sequencing with paired TCRαβ sequencing of T-cells as part of the study previously published by Huuhtanen et al. [30], involving single-cell analysis of immune recognition in chronic myeloid leukemia patients following tyrosine kinase inhibitor discontinuation. Processed gene-expression matrices generated using Cell Ranger (v3.0.0) with the GRCh38 reference genome and default parameters, along with the associated metadata, were obtained directly from the authors and are publicly available via Zenodo (accession: 10.5281/zenodo.7330586) [30]. Single-cell RNA-seq data were provided as 10x Genomics gene-count matrices with accompanying per-cell metadata, including patient identity, sampling timepoint, batch, and clinical outcome category. Raw counts were imported into an AnnData object, and standard quality-control procedures were applied to retain high-quality transcriptomes. Cells expressing fewer than 200 genes or exhibiting high mitochondrial RNA content (>10% of total UMI counts) were removed to exclude low-quality, apoptotic, or damaged cells. Following filtering, gene-expression values were library-size normalized and log-transformed to mitigate differences in sequencing depth. All batch annotations (patient, sample, and timepoint) were retained within the metadata to enable integrative analyses and systematic comparison of transcriptional programs across treatment outcomes (TFR versus early and late relapse).

The GAFA-based pipeline for single-cell analysis comprises five major steps (Figure 1): (1) preprocessing of scRNA-seq data; (2) inference of NK cell developmental states; (3) generation of predictive gene panels; (4) construction of gene modules; and (5) validation of candidate gene modules. At the conclusion of each major step, we produce corresponding analytical outputs, including annotated NK cell clusters, trajectory-associated differentially expressed genes (DEGs), optimized predictive gene panels, transcription factors (TFs) and their regulatory modules, as well as novel gene signatures with functional annotations. To ensure analytical rigor and result quality, we applied quantitative evaluation metrics at each stage: batch mixing scores to assess batch-effect correction; statistical significance thresholds based on *p*-values and log fold changes for DEG identification; accuracy and area under the curve (AUC) for gene panels, with genes prioritized according to their frequency of selection across validation folds to guide gene panel optimization; and Gene Ontology (GO) enrichment *p*-values to identify functionally relevant TFs and regulatory modules. The GAFA-based predictive gene panel was validated through systematic support from PubMed literature.

### 2.2. Cell Clustering and Cell Stage Identification

To integrate multiple samples and correct for batch effects, we employed the scVI-tools framework [31]. It was used to learn a joint latent representation of the data while accounting for batch differences. Briefly, scVI models the gene expression matrix using a variational autoencoder, capturing biological signals in a low-dimensional latent space while regressing out batch-specific effects. The model was trained using each cell’s batch (sample ID) as a known covariate, yielding a d-dimensional latent embedding for each cell (d = 20). This batch-corrected latent space was then used as the basis for visualization and clustering. A Uniform Manifold Approximation and Projection (UMAP) [32] was computed from the scVI latent embeddings to visualize cells in 2D.

Unsupervised clustering was performed in the latent space by constructing a k-nearest neighbors graph (k = 15) and applying the Leiden community detection algorithm [33]. An initial clustering at a moderate resolution (resolution = 1.0) yielded distinct clusters that corresponded to significant immune cell populations in the dataset.

NK cells were identified within these clusters using automated cell-type annotation and marker gene analysis. We utilized CellTypist [34], a machine learning-based tool for rapid cell type classification, to assign provisional identities to each cluster. Clusters annotated as NK cells by CellTypist and confirmed by the expression of canonical NK cell markers such as *NCAM1* and *FCGR3A* were extracted for a focused sub-analysis. All cells belonging to these NK-designated clusters were subset from the full dataset. This NK cell subset was then analyzed separately to resolve finer-grained heterogeneity. Clustering (resolution = 0.3) was repeated on the NK-only data. A new UMAP visualization was generated for NK cells alone to delineate NK subpopulations. This approach yielded 6 NK cell clusters (subtypes), which were retained for downstream analyses. The identity of these NK subclusters was validated by examining known NK subtype markers. Cell cycle scores were calculated to ensure they did not affect integration.

### 2.3. Pseudotime Trajectory Inference

To characterize developmental and activation trajectories within NK cell populations, we performed diffusion pseudotime (DPT) analysis. A diffusion map was constructed from the NK cell expression manifold, modeling cellular transitions through a graph of transcriptional similarity. Based on cluster composition and canonical marker patterns, two major NK cell lineages were hypothesized. CD56^bright^ NK cells, representing the least mature and most transcriptionally naïve population on par with the previous publications [30,35,36,37,38], were therefore selected as the root state for both trajectories. Diffusion pseudotime values were then computed for all NK cells, ordering them along a continuum from the root toward terminal effector or memory-like end states.

Branch occupancy and trajectory positioning were compared between TFR and relapse groups by quantifying the distribution of cells along pseudotime. Differences in pseudotime distributions were assessed using the Mann–Whitney U test, with global comparisons across outcome categories evaluated using the Kruskal–Wallis test. Per-sample branch occupancy was also examined using Mann–Whitney U tests. These analyses enabled assessment of whether NK cells from TFR versus relapse patients preferentially populated distinct branches or progressed to different extents along shared developmental pathways.

### 2.4. GAM-Based Identification of Dynamic NK Genes and Gene Panel Derived from Random Forest

After defining the two NK cell lineages from the pseudotime analysis (L501: maturation, L503: cytotoxic), we next quantified gene expression dynamics along each trajectory. For each lineage separately, we restricted to NK cells assigned to that lineage and used the corresponding diffusion pseudotime values (pseudotime_501 or pseudotime_503) as a continuous covariate. Gene expression was modeled with a generalized additive model of the form expression g∼s(pseudotime), where s(⋅) is a spline term capturing smooth changes along pseudotime (linearGAM implementation) [39]. For each gene and lineage, the model returned a normalized association score (“score_norm”) summarizing the strength of pseudotime dependence. Genes with positive score_norm were retained as lineage-dynamic genes, and we defined a pooled pseudotime gene set as the union of dynamic genes from both lineages (GAM_501 ∪ GAM_503). This ensured that we captured genes engaged along either the maturation (L501) or cytotoxic (L503) trajectory. We further expanded this set by adding a small number of NK transcription factors that were also identified as dynamic in both lineages.

For classifier construction, features were z-scored, and we trained a random forest classifier (500 trees, balanced class weights, we implemented stratified cross-validation at the patient level by holding out entire patient samples during testing to prevent cell-level leakage). Specifically, models were trained on three relapse (either early relapse or late relapse) and one control samples and evaluated on the remaining one relapse and one control samples, resulting in eight unique train–test validation combinations. The classification analyses included paired samples collected at TKI discontinuation (baseline) and either at relapse or during early treatment-free remission (TFR). Single-cell data were restricted to clusters 0, 1, 3, and 5 across both lineages (GAM_501 ∪ GAM_503) to ensure consistent cell-state representation in model training and evaluation. Because the dataset comprises repeated single-cell measurements from a small number of samples, individual cells were treated as observations of transcriptional level. Accordingly, statistical tests and classifier performance reflect cell-level gene signatures and represent an exploratory, hypothesis-generating distinction between TFR and relapse samples. Model performance was evaluated using accuracy and ROC–AUC. Variable importance was quantified using the random forest feature importance scores (mean decrease in impurity), which we used to rank genes. To identify predictive signatures, we selected the top k ranked genes (k = 15) based on feature importance derived from the random forest model. Genes were then prioritized according to their selection frequency across the eight train–test validation combinations, ranking them by the proportion of folds in which they appeared within the top k feature panel.

### 2.5. Gene Regulatory Network Inference

GRN inference was performed using an integrated pipeline combining prior knowledge from transcription factor (TF) and protein–protein interaction (PPI) databases with the message-passing algorithm SCORPION [3]. A single global GRN was first constructed using the entire NK cell transcriptomic dataset, encompassing all identified clusters, to ensure the coverage of NK cell regulatory relationships. From this global network, condition-specific (TFR and relapse) and cluster-specific subnetworks were subsequently extracted. The prior network supplied to SCORPION integrated multiple evidence sources. Experimentally supported TF–target relationships were curated from MSigDB [40], DoRothEA [41], and TRRUST [42], while high-confidence PPIs were retrieved from the STRING database [43]. Only interactions with a combined STRING score ≥ 700 (high-confidence evidence) were included in the analysis. SCORPION was executed using a controlled learning rate (alpha = 0.05), L2 regularization term (lambda = 0.1), with 150 iterations.

Edges were retained if the absolute regulatory weight exceeded 0.3, corresponding to the top 1.5% of all weighted interactions. This filtering captured high-confidence TF–target relationships and yielded networks containing approximately 5000–8000 edges per condition.

PageRank centrality was computed to identify key regulators; TFs within the top 10th percentile of PageRank scores were designated as high-centrality regulators. Out-degree and betweenness centralities were also computed to assess regulatory breadth and control over information flow. The resulting directed graphs were imported into Cytoscape v3.8.0 [44] for visualization and topological analysis. To delineate co-regulated modules, the Markov Cluster Algorithm [45] was applied with an inflation parameter = 2.2, producing modules typically containing 20–40 genes.

### 2.6. Curation of NK Cell–Relevant Gene Annotation and Literature-Supported Evidence Table

To provide a context for the machine learning–derived NK cell gene panels and support biological interpretation, we constructed a literature-annotated gene evidence table for all genes included in the 18-gene panel. For each gene, published evidence was systematically curated from the PubMed database (https://pubmed.ncbi.nlm.nih.gov (accessed on 30 December 2025)). When direct NK-specific evidence was limited, studies establishing broader immune or inflammatory roles relevant to NK cell function were included to provide contextual support.

## 3. Results

### 3.1. Six Transcriptionally Distinct NK Cell States Span Activation, Maturation, Trafficking, and Immunoregulation

**Figure 2:**
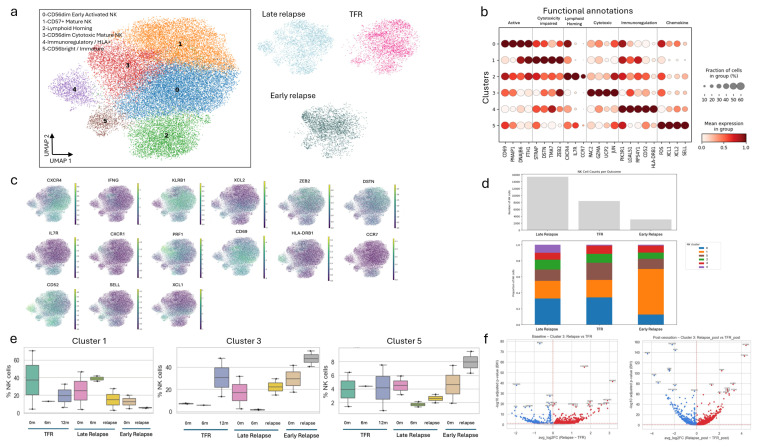
Identification of NK cell subtypes and their associations with clinical outcomes. (**a**) UMAP visualization of re-clustered NK cells annotated by NK subtypes and stratified by clinical outcomes (TFR, early relapse, and late relapse). Cluster numbers indicate Leiden cluster IDs. The right panel shows the latent distribution of NK cells across the three clinical outcome groups. (**b**) Marker-gene feature plots and dot plots defining distinct NK cell subsets. The x-axis denotes marker genes grouped by functional annotation, and the y-axis represents Leiden-identified NK cell clusters. (**c**) Single-cell expression landscapes of representative NK subtype marker genes projected onto the UMAP embedding. (**d**) Mean fraction of NK cell clusters per patient across clinical outcome groups. The upper bar plot shows the total number of cells per outcome group, while the lower stacked bar plot illustrates cluster composition and relative abundance across outcomes. (**e**) Boxplots comparing cluster frequencies across clinical groups, highlighting statistically significant differences. (**f**) Volcano plots showing differential gene expression in cytotoxic NK cells at baseline and post-cessation.

The scRNA-seq revealed six distinct NK cell states across activation, maturation, trafficking, and immunoregulatory programs. Re-clustering of NK cells within the scVI-derived latent space resolved six robust transcriptional states (clusters 0–5), each forming discrete structures on the NK-only UMAP embedding (Figure 2a,b). Marker-gene profiles delineated functional identities (Figure 2c and Table S1). Cluster 0 expressed *CD69* (logFC = 1.1, adjusted *p*-value < 0.01), *PMAIP1* (logFC = 4.6, adjusted *p*-value < 0.01), *DNAJB6* (logFC = 2.4, adjusted *p*-value < 0.01), and cytotoxic effector molecules, consistent with CD56^dim^ early activated NK cells. Cluster 1 was enriched for *STRAP* (logFC = 2.0, adjusted *p*-value < 0.01), *DSTN* (logFC = 3.0, adjusted *p*-value < 0.01), *TMA7* (logFC = 2.0, adjusted *p*-value < 0.01), and *ZEB2* (logFC = 2.4, adjusted *p*-value < 0.01), characteristic of CD57+ terminally mature CD56^dim^ NK cells. Cluster 2 showed high *CXCR4* (logFC = 2.6, adjusted *p*-value < 0.01), *IL7R* (logFC = 2.4, adjusted *p*-value < 0.01), and *CCR7* (logFC = 3.9, adjusted *p*-value < 0.01), defining a lymphoid-homing or trafficking phenotype with reduced immediate effector potential. Cluster 3 upregulated *RAC2* (logFC = 2.2, adjusted *p*-value < 0.01), *GZMA* (logFC = 2.7, adjusted *p*-value < 0.01), *UCP2* (logFC = 1.5, adjusted *p*-value < 0.01), and *JUN* (logFC = 2.1, adjusted *p*-value < 0.01), corresponding to CD56^dim^ cytotoxic NK cells with strong inflammatory and effector activity. Cluster 4 exhibited prominent *PIK3R1* (logFC = 1.8, adjusted *p*-value < 0.01), *LGALS1* (logFC = 2.8, adjusted *p*-value < 0.01), *RPS4Y1* (logFC = 1.6, adjusted *p*-value < 0.01), *CD52* (logFC = 3.5, adjusted *p*-value < 0.01), and *HLA-DRB1* (logFC = 5.6, adjusted *p*-value < 0.01) expression, identifying an HLA-DR^+^ immunoregulatory NK subset with transcriptional enrichment for antigen presentation and interferon-responsive pathways. Finally, cluster 5 expressed *XCL1* (logFC = 4.4, adjusted *p*-value < 0.01), *XCL2* (logFC = 3.4, adjusted *p*-value < 0.01), *SELL* (logFC = 4.0, adjusted *p*-value < 0.01), and *FOS* (logFC = 2.2, adjusted *p*-value < 0.01), consistent with a CD56^bright^-like, cytokine-responsive population. Together, these six states form a continuum extending from chemokine-producing CD56^bright^-like NK cells (cluster 5), through early activation (cluster 0), to several distinct end-states terminal maturation (cluster 1), lymphoid trafficking (cluster 2), cytotoxic effector function (cluster 3), and HLA-DR^+^ immunomodulation (cluster 4).

NK cell subset composition diverges between treatment-free remission and relapse in the quantification of NK cell subset distributions across patients and timepoints (Figure 2a and Figure S1). Across all samples, cluster 0 represented the predominant NK population. TFR samples, both at baseline and during follow-up, were dominated by cluster 0, with modest contributions from cluster 3 and a near-absence of cluster 4, indicating a repertoire enriched for activated yet predominantly HLA-DR-NK cells. By contrast, late-relapse samples, particularly from patient 2, demonstrated substantial expansion of cluster 4 alongside increased cluster 3 representation (Figure S1). The cluster-4 gene-module score in patient 2 showed a modest decrease from baseline to relapse (*p* = 0.033) despite the increased abundance of cluster-4 cells, suggesting that relapse reflects both quantitative expansion and qualitative transcriptional remodeling within the HLA-DR^+^ subset. Comparisons of baseline samples revealed that the cluster-4 signature was markedly elevated in late-relapse relative to TFR baselines (*p*-value < 0.01), with early relapse patients exhibiting intermediate levels. These findings indicate that an HLA-DR^+^ bias is already present at the time of TKI discontinuation in patients who later undergo late relapse.

Averaging NK cell fractions across all timepoints per patient (Figure 2d) further highlighted outcome-specific patterns. TFR patients (P5, P6) displayed the highest burden of cluster 0 cells and virtually no cluster 4 representation. Late-relapse patient 2 showed the greatest proportion of cluster 4 and intermediate proportions of clusters 1 and 3. Early relapse patient 7 exhibited a pronounced expansion of cluster 3 (cytotoxic CD56^dim^ NK) with moderate levels of clusters 0 and 5, suggesting a repertoire skewed toward cytotoxic effector phenotypes. Boxplots summarizing cluster abundances by outcome group and clinical timepoint (Figure 2e) revealed additional trends. Cluster 1 (CD57+ mature NK) showed the highest median abundance at TFR baseline, with reduced levels in relapse groups at relapse (Kruskal–Wallis *p* < 0.05), supporting a modest, non-significant enrichment of CD57^+^ NK cells in individuals who sustain long-term TFR. Cluster 3 (cytotoxic CD56^dim^ NK) expanded over time in all groups, with the most marked increase at relapse, particularly early relapse, where it constituted ~40–45% of NK cells. Cluster 5 (CD56^bright^-like) remained a minor population throughout.

Cytotoxic NK cells exhibited greater transcriptional plasticity in TFR than in relapse. To assess whether NK cell adaptation differs between clinical outcomes within a shared functional state, we compared transcriptomes of cluster-3 cytotoxic CD56^dim^ NK cells at baseline and following TKI discontinuation (Figure 2f). At baseline, transcriptional differences between TFR and relapse were relatively modest. TFR samples showed elevated expression of ribosomal genes (*RPS26*, *RPS6*, *RPL14*) and transcription factors *FOS* and *KLF6*, whereas relapse samples exhibited higher expression of *LGALS9B*, *MYOM2*, *IFITM3*, and several ribosomal genes (*RPL41*, *RPL26*, *EEF1A1*). Both groups nonetheless displayed similar cytotoxic signatures dominated by *GZMA* and related effector molecules, indicating broadly comparable basal killing potential. After TKI cessation, however, cluster-3 NK cells diverged sharply between outcomes. Relapse samples showed coordinated downregulation of stress-response genes, including *RHOB*, *KLF2*, and *PPP1R15A*, suggesting impaired adaptive capacity. In contrast, TFR samples upregulated cytotoxic and signaling molecules including *JAK1*, *FCER1G*, and *TXNIP*, consistent with enhanced effector function and stress-adaptation responses. *LGALS9B* and *MYOM2* remained persistently elevated in relapse, reinforcing a distinct, relapse-biased transcriptional pattern. Quantitatively, cytotoxic NK cells from TFR patients exhibited far more extensive transcriptional remodeling after drug withdrawal than those from relapsing patients (553 vs. 64 differentially expressed genes, FDR < 0.05). These findings indicate that cytotoxic NK cells in TFR undergo a broad network difference of effector and stress-response programs. In contrast, cells from relapsing individuals remain comparatively static, failing to mount the adaptive response that may be required for sustained immunological control of residual leukemia.

### 3.2. Pseudotime Analysis Reveals Outcome-Specific States Along NK Cell Differentiation Trajectories

**Figure 3:**
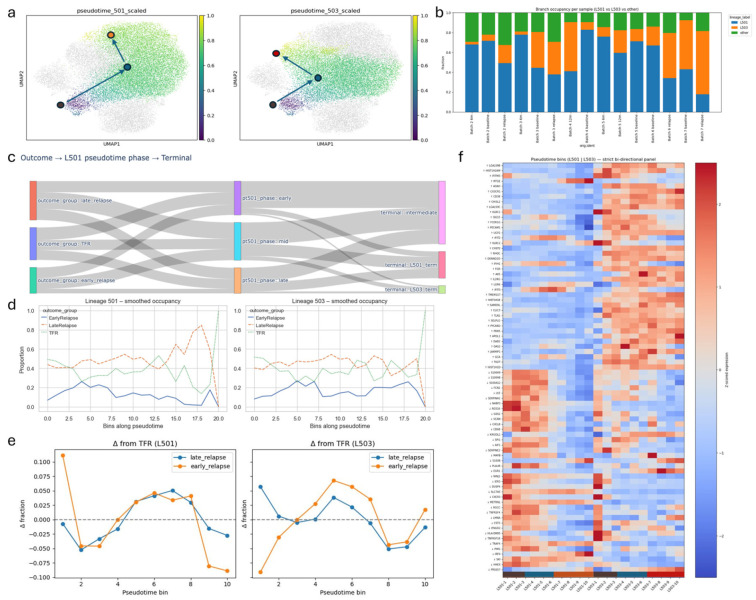
NK cell differentiation trajectories across clinical outcomes. (**a**) UMAP embeddings annotated with lineage-specific pseudotime for the maturation (L501) and cytotoxic (L503) NK cell trajectories. (**b**) Branch occupancy proportions across individual samples for each lineage. (**c**) Sankey diagrams illustrating the contributions of clinical outcome groups (TFR, early relapse, and late relapse) to pseudotime phases and terminal lineage states. (**d**) Smoothed lineage occupancy profiles along pseudotime, stratified by clinical outcome. (**e**) Differences in branch occupancy relative to TFR across pseudotime bins for the maturation (L501) and cytotoxic (L503) lineages in CML NK cells in dot plots. (**f**) Bidirectional pseudotime heatmap showing dynamic gene expression patterns along the L501 and L503 trajectories.

To reconstruct NK cell developmental dynamics, we applied diffusion pseudotime (DPT) analysis, anchoring trajectories in cluster 5 (CD56^bright^-like NK cells), which expressed *XCL1*, *XCL2*, *SELL* and *FOS*. DPT revealed two principal lineages emerging from this root state (Figure 3a–c).

The first trajectory (L501: 5 → 0 → 1) progressed through early activated cluster 0 toward cluster 1, consistent with a canonical maturation path culminating in CD57+ NK cells. The second trajectory (L503: 5 → 0 → 3) shared the same intermediate state but terminated in cluster 3, representing differentiation into cytotoxic effector NK cells. UMAPs coloured by lineage-specific pseudotime demonstrated smooth, continuous gradients along each branch.

Analysis of branch occupancy uncovered outcome-associated skews (Figure 3d). TFR samples exhibited a balanced representation across both lineages, maintaining substantial occupancy throughout pseudotime along L501 and L503. Early relapse samples showed markedly reduced occupancy of L501, typically <20% across pseudotime, and an overall depletion of cells assigned to either lineage, with only a modest enrichment toward the cytotoxic L503 branch. Late-relapse samples displayed an intermediate phenotype: contributions to both branches were detectable, but with elevated occupancy of L503 and a progressive rise in L501 occupancy toward terminal pseudotime.

Smoothed occupancy curves further quantified these trends. Along L501, TFR trajectories remained stable, early relapse trajectories were consistently suppressed, and late-relapse trajectories gradually increased toward terminal states. Along L503, late-relapse samples showed higher occupancy than TFR across most of pseudotime, whereas early relapse samples remained below TFR in nearly all bins. Sankey diagrams linking outcome group, pseudotime phase, and terminal state (Figure 3e) confirmed that TFR trajectories predominantly reached L501 intermediates or termini, whereas early relapse trajectories disproportionately accumulated in the L503 terminal state. Late-relapse trajectories bifurcated between both outcomes.

A pseudotime-ordered heatmap of dynamic genes (Figure 3f) contextualized these trajectories. Early pseudotime bins at the CD56^bright^-like root expressed *XCL1*, *XCL2*, *SELL*, and *FOS*, reflecting chemokine-secreting and cytokine-responsive phenotypes. Transition through cluster 0 involved induction of *CD69* and *PMAIP1*, marking early activation. Terminal states diverged sharply: late L501 bins were enriched for maturation-associated regulators such as *ZEB2*, whereas late L503 bins up-regulated cytotoxic and inflammatory effectors, including *GZMA*, *RAC2*, *JUN*, *UCP2*, and *LGALS1*.

Together, these findings indicate that durable TFR is characterized by balanced engagement of both maturation and cytotoxic NK cell trajectories based on the trajectories L503 and L501. In contrast, relapse, especially early relapse, is marked by impaired progression along the CD57+ maturation branch and preferential accumulation in cytotoxic end states.

### 3.3. ML-Derived Cell-Level Predictive Panels of 18-Gene Signatures

Pseudotime-resolved gene dynamics distinguish maturation- and cytotoxic-biased NK trajectories. Lineage-specific generalized additive modeling (linearGAM) along the L501 (maturation) and L503 (cytotoxic) branches identified several hundred genes whose expression varied smoothly with pseudotime in each trajectory. Taking the union of dynamic features across both lineages yielded a pooled pseudotime-responsive NK gene set, ensuring that genes active along either developmental axis were retained rather than restricting the analysis to a single branch. At the single-gene level, several features displayed strong transcriptional changes associated with clinical outcome. *MALAT1*, *HLA-C*, and *IFITM1* were consistently enriched in relapse samples (log_2_FC ≈ +0.7), whereas classical TFR-associated transcripts including *S100A8*, *S100B*, *LYZ*, and *TSC22D3* were markedly depleted in relapse and enriched in TFR NK cells (log_2_FC ≈ −0.8 to −1.7), consistent with their established roles in anti-inflammatory and tissue-repair phenotypes.

Outcome-associated genes show distinct coupling to the two NK lineages. To quantify how these outcome-associated genes align with NK developmental trajectories, we computed the Spearman correlation between expression and pseudotime within each lineage. Relapse-high genes demonstrated pseudotime coupling in both branches (mean ρ_501_ ≈ 0.80; mean ρ_503_ ≈ 0.52), indicating that relapse-associated programs are progressively engaged along both maturation and cytotoxic differentiation. In contrast, TFR-high genes maintained strong dynamics only along the L501 maturation branch (mean ρ_501_ ≈ 0.68) and were essentially flat across the L503 cytotoxic trajectory (mean ρ_503_ ≈ 0.05). These relationships indicate that relapse-associated gene programs preferentially track the cytotoxic L503 axis, whereas TFR-associated features remain tightly linked to the maturation continuum.

To determine whether outcome-related transcripts could serve as a predictive classifier, we trained a random forest model using NK cells labeled as TFR or relapse and ranked features by importance. We identified 18 genes that appeared in at least 50% of the top-15 ranked gene panels across the eight train–test validation combinations (Table 1 and Table S2). Among these, *HLA-C* and *GNLY* were the most consistently selected genes, each appearing in 75% of validations. A second group—including *IFITM2*, *NFKBIA*, *FCER1G*, *TSC22D3*, *IFITM3*, *CST7*, *MT2A*, *GZMA*, *PIM1*, *ACTB*, *S100A8*, *CYBA*, *S100B*, *MALAT1*, *MYOM2*, *and LYZ*—were selected in 50% of folds. This recurrent selection pattern suggests that interferon-responsive transcripts and cytotoxic effector genes provide complementary predictive information beyond the core signatures, exploratorily separating TFR from relapse phenotypes.

The biological composition of the classifier-derived panels is consistent with known axes of NK cell function and immune activation (Table 2). Five out of the 18-gene panel are conventional with well-established, canonical roles in NK cell identity, cytotoxic function, or immune recognition, widely cited across NK literature and often used as markers. *CST7* has been implicated as a key mediator within the tumor microenvironment, where it modulates NK cell cytotoxicity and thereby influences the effectiveness of antitumor immune responses [46,47]. *FCER1G* (*FcRγ*) encodes a key signaling adaptor for Fc receptors and NK cell killer activating receptor (KAR) complexes, is highly expressed in NK cells, and plays an essential role in regulating NK cell activation and effector function [48,49,50]. *GNLY* is consistent with a cytotoxic effector program aligned with NK functional state differences relevant to outcome [51,52,53]. *GZMA* encodes a natural killer cell-associated serine protease that functions as a critical effector molecule required for target cell lysis by both cytotoxic T lymphocytes and natural killer cells [54,55]. *HLA-C* reflects MHC class I allelic variation that modulates NK cell education through KIR interactions, thereby shaping NK functional tuning and compensatory immune surveillance [39,40]. *ACTB* has been implicated in NK cell function, as a *de novo G342D* mutation in *ACTB* disrupts actin cytoskeletal dynamics, leading to functional NK cell deficiency characterized by increased cell spreading, impaired lytic synapse termination, and reduced cytotoxic capacity [56]. *CYBA* has been implicated in immune regulation, as analysis of lymphocyte populations demonstrated that *Cyba^tm1a^* mice exhibited an increased proportion of activated T cells and NK cells in the lungs compared with controls, highlighting its potential role in modulating immune responses during metastatic colonization [57]. The IFITM-family features (*IFITM2/3*) support an interferon-primed state that may accompany chronic antigen exposure and inflammatory activation [58,59,60]. The appearance of *LYZ*/*S100A8*/*S100B* genes more typical of myeloid inflammation may indicate an inflammation-coupled immune milieu associated with relapse outcome or reflect ambient-RNA/doublet sensitivity in single-cell assays; we therefore treat these signals as hypothesis-generating and interpret them in conjunction with NK-state markers, trajectory position, and GRN context [61,62,63,64]. Several higher-ranked “non-canonical” features may reflect broader regulatory or stress programs that intersect with cytotoxic lymphocyte biology, including *MALAT1*-associated transcriptional regulation [65] and stress-response pathways involving metallothionein (*MT2A*) [66]. *MYOM2*, while not a canonical NK marker, has been reported in blood-NK transcriptional signatures in disease contexts and may capture an outcome-linked NK state component rather than a direct effector mechanism [67]. *NFKBIA* has been associated with a highly inflammatory CD56^dim^ CD16^high^ NK cell subset characterized by elevated expression of *CCL3*, *CCL4*, and *CCL4L2*, indicating an enhanced capacity to recruit additional immune cells, including T cells [28]. *PIM1* encodes a serine/threonine kinase that operates downstream of IL-15/JAK–STAT signaling in NK cells, where it promotes cytokine-driven survival, proliferation, and metabolic fitness, thereby supporting NK cell functional competence [68]. High *TSC22D3* and low *GBP1* expression may be associated with early hepatocellular carcinoma recurrence after liver resection through immunosuppressive mechanisms [69].

### 3.4. Outcome-Specific Transcription Factor Networks Distinguish TFR from Relapses

**Figure 4:**
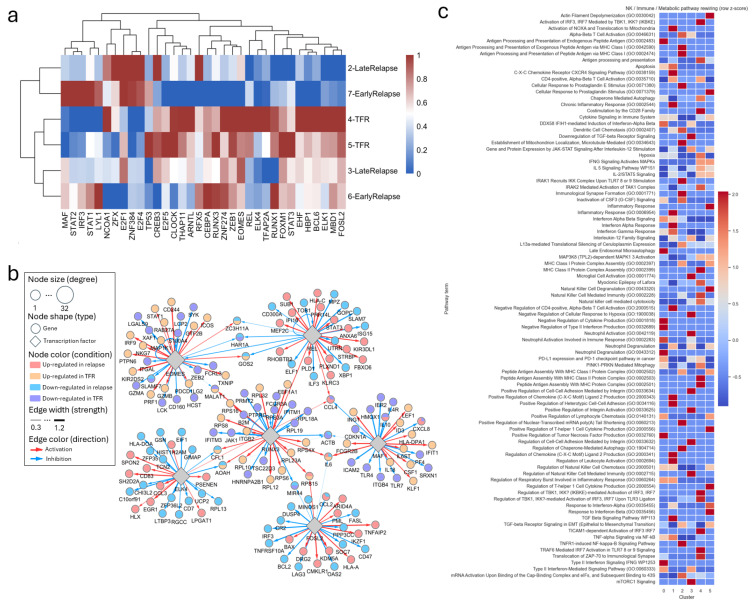
Regulon activity and outcome-associated gene regulatory networks with enriched biological functions. (**a**) Differential regulon activity along NK cell differentiation trajectories across clinical outcome groups. The heatmap represents the min–max scaled TF activities. (**b**) Outcome-stratified NK cell gene regulatory network highlighting key transcriptional regulators and their target genes. (**c**) Immune- and metabolism-related pathway enrichment across NK cell clusters.

To identify upstream regulators of NK cell programs associated with clinical outcome, we inferred a global GRN using SCORPION and quantified regulon activity at both pseudobulk and cluster resolution with 34 TFs (Figure 4a and Table S3). TFs selected for detailed analysis, the six TF encoded genes, *EOMES*, *ELK4*, *REL*, *FOSL2*, *RUNX3* and *MAF,* were chosen from linearGAM prefiltered dynamic genes from lineage 501 and 503 and based on high network centrality and outcome-associated patterns in regulon heatmaps.

In pseudobulk analyses, TFR and late-relapse samples exhibited stronger activity of differentiation- and interferon-linked regulons (*RUNX1*, *CEBPA*, *STAT1/2/3*) than early relapse samples, consistent with preserved maturation and cytokine-responsiveness programs. At the cluster level, these regulons were most active in clusters 0 and 1, mirroring the abundance of early activated and CD57^+^ NK cells in TFR patients. In contrast, *ELK4*- and *REL*-associated regulons were enriched in cluster 4 and parts of cluster 0, corresponding to the immunoregulatory, HLA-DR^+^ NK subset expanded in relapse. Outcome-stratified network diagrams (Figure 4b and Tables S4 and S5) revealed extensive rewiring of TF–target relationships between TFR and relapse. Yellow nodes denote TFR-upregulated genes, green nodes genes reduced in relapse, brown nodes genes downregulated in TFR, and grey nodes relapse-upregulated genes. 

The *RUNX3* hub contained numerous TFR-up or relapse-down targets, including *PRF1*, *JAK1*, *CFL1*, *PTPRC*, *FCGR2A*, *RPL19*, and *EEF1A1*, indicating that *RUNX3*-associated targets include genes involved in perforin-mediated cytotoxicity, cytokine signaling, cytoskeletal remodeling, and efficient translation in TFR. Conversely, relapse-up *RUNX3* targets (e.g., ribosomal genes *RPS4X*, *RPL23A*, *RPL32*, *RPL16*, *RPS6* and antiviral genes *IFITM1*, *RPS3A*) suggest a shift toward a translation-heavy, interferon-primed state in relapse. The *EOMES* module also displayed outcome-specific partitioning: TFR-up targets (*STAT1*, *CD8B*, *ITGAL*, *CX3CR1*) aligned with mature cytotoxic NK programs, whereas relapse-up targets (*NAP1L1*, *IRF9*, *ZEB2*) indicated an interferon-loaded, less cytotoxic configuration. Similarly, *ELK4* supported differentiation and transcriptional feedback pathways in TFR (*AOAH*, *ZFP36L2*, *HLX*, *CD7*), but favored chemokine-rich, adhesive programs in relapse (*CCL3*, *SPON2*). The *REL* hub further separated TFR and relapse states. TFR-up targets (*ZC3H12A*, *TOB1*, *ILF3*, *STRBP*, *PRR14L*, *ELF1*) were linked to RNA stability and signaling restraint. In contrast, relapse-up targets (*HLA-C*, *SLAMF7*, *XBP1*, *ISG15*, *ITK*) indicated redirection of non-canonical NF-κB signaling toward a *SLAMF7*^+^, interferon-responsive phenotype. For *FOSL2* and *MAF*, outcome specificity was most apparent in GRN topology. The *FOSL2* hub was heavily relapse-biased, with targets including *IL6*, *CCL2*, *UCP2*, *IRF1*, *DRG2*, *ARID4A* and multiple ribosomal genes, whereas negative regulators (*SOCS7*, *CD47*, *DUSP1*) were reduced in relapse. The *MAF* regulon showed strong divergence: TFR-up targets (*HLA-DMB1*, *TLR7*, *LEF1*, *CSF1*, *CXCL9*) formed an antigen-presentation and T cell-recruitment module, whereas relapse-up targets (*IL1B*, *IL18*, *IFIT1*, *FCGR2B*, *ICAM1*) formed an inflammatory, antiviral-sensor and inhibitory-receptor program. 

Together, these GRN analyses indicate that TFR NK cells preferentially engage *RUNX3*–*EOMES*–*ELK4*–*REL* regulatory modules that reinforce cytotoxicity, antigen presentation, cytokine responsiveness, and post-transcriptional control. Relapse NK cells instead rely on *FOSL2*–*MAF*-dominated programs enriched for inflammatory mediators, translational machinery, and inhibitory receptors, reflecting a shift toward dysregulated or exhausted NK states accompanying molecular recurrence. 

### 3.5. Pathway Enrichment and GO Reveals Functional Specialization Across NK States

Pathway and Gene Ontology enrichment analysis of cluster-specific upregulated genes (Figure 4c) showed functional differences along the NK cell spectrum. Cluster 0 (early activated CD56^dim^ NK) was enriched for actin remodeling, apoptosis, chaperone-mediated autophagy, co-stimulation via CD28, and JAK–STAT signaling after interleukin 12 (IL-12) stimulation, consistent with an early activation state poised for effector function rather than a fully inflammatory end state. Cluster 1 (CD57^+^ mature CD56^dim^ NK) showed selective enrichment for positive regulation of integrin activation, heterotypic cell–cell adhesion, and lymphocyte chemotaxis, fitting with stable target engagement and late effector function. Cluster 2 (lymphoid-homing/trafficking NK) was specifically enriched for CX-C chemokine receptor CXCR4 signaling, chemokine ligand production, and leukocyte migration terms, supporting a migratory role with limited immediate cytotoxicity. Cluster 3 (cytotoxic CD56^dim^ NK) displayed the broadest inflammatory and stress-response program, with strong enrichment for inflammatory response, interferon-α/β and interferon-γ responses, IL-6 and IL-23/STAT3 signaling, TLR/IRAK1–IKK pathways, TNF-α/NF-κB signaling, TGF-β signaling, and mTORC1 signaling, matching its designation as an inflammatory effector subset. Cluster 4 (HLA-DR^+^ immunoregulatory NK) showed the highest enrichment for antigen processing and presentation via MHC class I and II, peptide antigen assembly into MHC complexes, interferon-α signaling, and PD-1/PD-L1 checkpoint–related pathways, consistent with an APC-like, interferon-responsive NK population. Finally, cluster 5 (CD56^bright^-like NK) was enriched for regulation of NK cell chemotaxis and NK cell mediated immunity, regulation of leukocyte activation, Th1-type cytokine production, and lymphocyte chemotaxis, indicating a cytokine-responsive, immunoregulatory progenitor-like compartment rather than a TGF-β/mTORC1-dominated state.

## 4. Discussion

After rigorous batch correction, the enhanced resolution of NK cell heterogeneity revealed six transcriptionally distinct states spanning activation, maturation, trafficking, cytotoxicity, immunoregulation, and a CD56^bright^-like progenitor compartment. This organization is broadly concordant with previous high-dimensional single-cell studies that have described human NK cell continua from CD56^bright^ cytokine producers to CD56^dim^ cytotoxic and tissue- or stress-adapted subsets [28,35,50,70]. Within this common framework, however, we observed outcome-specific skews. TFR samples were dominated by early activated CD56^dim^ NK cells (cluster 0) with comparatively little HLA-DR^+^ immunoregulatory signal (cluster 4). In contrast, late-relapse samples showed a marked expansion of HLA-DR^+^ NK cells and increased representation of cytotoxic cluster 3. Early relapse samples were particularly enriched for cytotoxic CD56^dim^ NK cells (cluster 3), consistent with prior observations that NK cell activation alone is not sufficient for durable disease control and can even herald impending dysfunction or exhaustion in chronically stimulated or leukemia-associated settings [50,71,72].

These compositional differences align with clinical studies showing that higher absolute NK cell counts and more mature CD57^+^/CD16^+^ NK subsets at TKI discontinuation are associated with improved TFR [23,24,26,73], but add another layer: it is not only the presence of cytotoxic NK cells that matters, but which transcriptional programs they engage after drug withdrawal. Our analysis of cluster-3 cytotoxic NK cells highlights this point: at baseline, TFR and relapse patients had broadly similar cytotoxic signatures, but following TKI cessation, TFR cells mounted extensive transcriptional remodeling involving mitochondrial, proteostasis, and stress-adaptation pathways, whereas relapse cells remained comparatively static and downregulated translational-stress genes. This suggests that the capacity of cytotoxic NK cells to reprogram their metabolism and stress responses in the face of renewed leukemic antigen exposure may be associated with sustained TFR, in line with broader work linking NK metabolic fitness to effective anti-tumor function [50,72].

Lineage-resolved pseudotime and NK trajectories together deciphered the cell-state-level differences between TFR and relapse. By anchoring diffusion pseudotime in CD56^bright^-like NK cells (cluster 5) and following differentiation through an early activated intermediate (cluster 0), we reconstructed two principal NK cell lineages: a maturation trajectory (L501; 5 → 0 → 1) culminating in CD57^+^ CD56^dim^ NK cells (cluster 1), and a cytotoxic trajectory (L503; 5 → 0 → 3) terminating in inflammatory, effector CD56^dim^ NK cells (cluster 3). These trajectories mirror previously described maturation and effector paths in human bone marrow and peripheral blood NK cells derived from single-cell and functional studies [35,50,70].

Outcome-stratified branch occupancy demonstrated that TFR patients maintained balanced engagement of both lineages, with stable occupancy across L501 and L503 pseudotime. Early relapse patients, by contrast, showed a pronounced depletion of the CD57^+^ maturation branch, with uniformly low L501 occupancy and preferential accumulation in the cytotoxic L503 terminal state. Late-relapse patients occupied an intermediate position, with elevated L503 occupancy and a gradual increase in L501 occupancy at later pseudotime. Taken together, these patterns support a model in which successful TFR is associated with both cytotoxic function and preserved progression along a maturation trajectory. In contrast, an overrepresentation of terminally cytotoxic states characterizes early relapse without adequate maturation buffering, and late relapse reflects a slow reshaping of this balance over time.

Pseudotime-informed gene selection links NK trajectories to clinical outcomes. To connect these trajectories to specific molecular features, we applied lineage-specific GAMs to identify genes whose expression changed smoothly with pseudotime along L501 and L503 and then prioritized a pooled pseudotime-responsive set. This strategy prioritized genes that were dynamically regulated along NK cell differentiation and associated with clinical outcomes.

At the single-gene level, we found a coherent split between relapse- and TFR-associated signals. *MALAT1*, *HLA-C*, and *IFITM1* were consistently higher in relapse (log_2_FC ≈ +0.7; single-gene AUC ≈ 0.70–0.74), aligning with a picture of interferon-primed, activation- and cytotoxicity-linked states that, when dysregulated, may predispose to exhaustion or ineffective surveillance. In contrast, *S100A8*, *S100A9*, *LYZ*, and *TSC22D3* were enriched in TFR NK cells (log_2_FC ≈ −0.8 to −1.7; AUC < 0.4 for relapse prediction), suggesting a counterbalancing role for myeloid-related, alarmin, and immunoregulatory programs that support durable NK function. Many of these genes, or their pathways, have been implicated in broader immune regulation, interferon signaling, or leukocyte homeostasis in other contexts [27,28,72], further supporting their biological plausibility as NK modulators in CML.

Importantly, the lineage analysis showed that these outcome-associated genes are not uniformly dynamic across the two trajectories. When we quantified the correlation between gene expression and pseudotime in each lineage, relapse-high genes showed strong dynamics along both L501 and L503 (mean ρ_501_ ≈ 0.80, mean ρ_503_ ≈ 0.52). In contrast, TFR-high genes remained strongly dynamic along the maturation branch (mean ρ_501_ ≈ 0.68) but were essentially flat along the cytotoxic lineage (mean ρ_503_ ≈ 0.05). This suggests that relapse-associated genes are preferentially engaged as NK cells progress toward the cytotoxic end state, whereas TFR-associated genes track more tightly with maturation dynamics. These findings provide a gene-level link between the trajectory imbalances observed in pseudotime and the clinical outcomes of TFR versus relapse.

AI-guided NK cell-level gene panels serve as data-driven predictive modules of relapse. Using the intersected set of pseudotime-dynamic, outcome-associated genes, we trained a random forest classifier to distinguish TFR from relapse at the single-cell level and ranked genes by importance. Several genes represent canonical NK markers with well-established roles in cytotoxicity and immune recognition, including *CST7*, *GNLY*, *GZMA*, *FCER1G*, and *HLA-C*, reflecting core effector machinery and NK education via KIR–MHC interactions. Additional features capture interferon-primed activation states (*IFITM2/3*), inflammatory signaling (*NFKBIA*-associated CD56^dim^ CD16^high^ subsets), and cytokine-driven survival pathways (*PIM1* downstream of IL-15/JAK–STAT). Other transcripts, such as *MALAT1*, and *MT2A*, likely represent broader regulatory and stress-response programs intersecting with NK functional states. The presence of *LYZ* and *S100*-family genes may indicate an inflammation-coupled immune milieu linked to relapse outcome or technical sensitivity inherent to single-cell assays; thus, these signals are interpreted cautiously within the context of NK-state markers, trajectory position, and regulatory network inference.

From a translational perspective, these panels should be viewed as hypothesis-generating, NK-focused biomarker candidates rather than ready-to-use clinical signatures. The 18-gene panel represents an expanded, higher-capacity signature for exploratory prediction tasks and mechanistic stratification. The fact that these panels were derived from pseudotime-aware, lineage-pooled genes strengthens the argument that they capture dynamic NK adaptation trajectories rather than static baseline differences alone.

Regulatory network difference distinguishes TFR- and relapse-associated NK programs. To place these gene-level differences into a regulatory context, we inferred a global NK cell GRN using SCORPION and examined TF–target structures stratified by outcome. Consistent with our prior expectations and with emerging literature on NK transcriptional control, PageRank-based centrality and regulon activity highlighted distinct TF modules associated with TFR versus relapse [50,70,72]. *RUNX3*, *EOMES*, *ELK4*, and *REL* formed part of a TFR-favoring axis, with targets enriched for perforin-mediated cytotoxicity (*PRF1*), cytokine signaling (*JAK1*, *STAT1*), adhesion and homing (*ITGAL*, *CX3CR1*), and post-transcriptional restraint (*ZC3H12A*, *TOB1*, *ILF3*). These features are consistent with a poised, cytotoxic, and cytokine-responsive NK state capable of sustained surveillance after TKI withdrawal [70,72].

By contrast, *FOSL2* and *MAF* hubs were heavily relapse-biased, with GRN modules enriched for inflammatory cytokines and chemokines (*IL6*, *CCL2*, *IL1B*, *IL18*), antiviral sensors (*IFIT1*, *ISG15*), inhibitory and Fc receptors (*FCGR2B*, *SLAMF7*, *ICAM1*), and translational machinery (ribosomal genes). This configuration resembles an interferon-loaded, inflamed, and inhibitory-receptor-rich state that may reflect chronic stimulation and evolving exhaustion, in line with broader work implicating AP-1 family factors and *MAF* in cytotoxic lymphocyte dysfunction and systemic inflammation [70,72,74,75]. Notably, several of the genes that emerged in our pseudotime- and outcome-informed panels (e.g., *HLA-C*, *IFITM1*, S100 family members) also appeared as targets within these GRN modules. By intersecting TF regulons with the pooled GAM/DEG gene sets and annotating each target as TFR_up or Relapse_up, we could show that the outgoing edges of these high-centrality regulators are themselves enriched for outcome-biased targets. This supports a model in which TFR is underpinned by *RUNX3*–*EOMES*–*ELK4*–*REL*-driven programs that integrate cytotoxicity, antigen presentation, and signaling control, whereas relapse is associated with *FOSL2*–*MAF*-dominated rewiring toward inflammatory, translationally heavy, and inhibitory states [70,72,74,75].

Our findings are consistent with and extend previous work linking NK cell competence to TFR after TKI discontinuation. Clinical correlative studies such as IMMUNOSTIM [26], EURO-SKI [24], and related discontinuation cohorts have shown that higher NK cell counts and more mature NK phenotypes (CD56^dim^, CD57^+^/CD16^+^) at baseline are associated with molecular relapse-free survival [23,24,26,73]. More recent single-cell studies have reported distinct NK subsets and functional signatures in CML patients who achieve TFR versus those who relapse but have remained mainly descriptive at the level of cluster frequencies or marker expression [28,29,30]. By integrating NK subsets, pseudotime trajectories, GRNs, and AI-based feature discovery in a single framework, our study suggests that three elements must be considered simultaneously: (i) which NK states are present, (ii) how they progress along maturation versus cytotoxic lineages, and (iii) which regulatory modules and gene programs are engaged along these paths. Within this combined view, TFR appears to reflect not merely an abundance of cytotoxic NK cells, but balanced occupancy of maturation and effector trajectories, coupled to specific *RUNX3*/*EOMES*/*ELK4*/*REL*-driven programs and a set of dynamically regulated genes that support sustained function. Relapse, especially early relapse, is instead associated with skewed occupancy toward cytotoxic end states, reduced maturation progression, and engagement of *FOSL2*/*MAF*-centered inflammatory and inhibitory networks.

Our study is subject to several limitations. The cohort is small (six patients, fifteen samples) and imbalanced across outcomes/timepoints, which limits statistical power and the stability of patient-level estimates. We mitigated this with patient-level pseudobulk contrasts and leave-patient-out checks, but the results remain hypothesis-generating rather than definitive biomarkers. As a result, statistical significance and classifier performance reflect cell-level organization and serve as exploratory hypothesis-driven prediction of TFR. We did not perform external validation, so all signatures, trajectories, and baseline classifiers should be viewed as preliminary until reproduced in an independent large and prospective dataset to establish patient-level predictive utility. GAFA does not rely on disease-specific training atlases or pre-labeled outcome cohorts, but instead integrates general transcription factor binding priors, curated protein–protein interaction networks [43], and unsupervised single-cell dynamics. Regulatory directionality and causal inference remain computationally inferred and not experimentally validated. These knowledge sources are organism-level and transferable across disease contexts, allowing the framework to operate even in settings with limited disease-specific annotation. However, in rare diseases or extremely small cohorts, the framework should be interpreted primarily as a hypothesis-generation tool rather than a turnkey predictive system. Under such conditions, regulatory modules and gene signatures provide structured mechanistic hypotheses that guide follow-up experiments and cross-cohort validation, rather than definitive clinical classifiers. As additional datasets become available, the GAFA can be iteratively refined, enabling progressive improvement in predictive and mechanistic resolution. TF activities and high-centrality regulators lack orthogonal support (like ATAC/CITE-seq or perturbation assays) [72,74,76]. Gene regulatory network inference revealed outcome-associated transcriptional programs rather than causal regulatory relationships. Functional validation is required to establish causality. The inferred regulons represent putative regulatory modules whose downstream targets are functionally linked to IFN-γ signaling, metabolic reprogramming, and immunoregulatory feedback circuits, providing testable hypotheses for future functional validation. Several genes prioritized in the predictive panels are not classical NK lineage markers but are instead associated with inflammatory signaling or immune microenvironmental interactions. These signals may reflect context-dependent NK-cell activation states, stress-responsive transcriptional programs, or microenvironmental influences captured at single-cell resolution rather than lineage contamination. Importantly, GAFA prioritizes genes based on trajectory dynamics, regulatory coherence, and predictive contribution, allowing identification of non-canonical yet outcome-informative features.

We plan to expand the cohort and perform external validation using independent CML discontinuation datasets; if available, we will also test concordance with bulk imatinib transcriptomes by deconvolution and baseline signature transfer. Prospective sampling with denser timepoints, inclusion of CITE-seq or scATAC-seq, and functional assays (flow cytometry of CD57^+^/CD16^+^/HLA-DR^+^, cytotoxicity, and cytokine release tests) will help ground the transcriptional states in protein and function.

## 5. Conclusions

In this study, we developed and applied an integrative GAFA pipeline to reanalyze single-cell NK cell transcriptomes from CML patients undergoing imatinib discontinuation, demonstrating that both the cellular composition and regulatory wiring of NK cell states are tightly associated with TFR versus relapse. By explicitly coupling latent-space representation learning, lineage-resolved pseudotime modeling, GRN inference, and AI-guided feature prioritization within a unified analytical framework, GAFA provides an integrated strategy that helps bridge the fragmented analytical components of conventional single-cell workflows. This integrative strategy moves beyond descriptive clustering to infer developmental trajectories, transcription factor-centric regulatory relationship, and outcome-predictive gene panels in an exploratory manner. Our analyses reveal that distinct NK cell differentiation trajectories and regulatory modules preferentially support durable immune control or predispose to relapse following TKI withdrawal, providing functional insight into NK cell-mediated surveillance in CML. Collectively, these findings establish GAFA as an integrative framework for uncovering single-cell gene signatures that support exploratory, hypothesis-driven prediction of TFR and inform immunologically guided treatment optimization.

## Figures and Tables

**Table 1:** The top predictive genes that appeared in the top 15 ranked features across eight validation folds. The “Count in top15” indicates the number of folds in which a gene was selected among the top 15 features, the “Number of validations” denotes the total number of validations evaluated (*n* = 8), and “Frequency” represents the proportion of folds in which the gene was selected.

Gene Symbol	Gene Name	Count in Top15	Number of Validations	Frequency
*GNLY*	Granulysin	6	8	0.75
*HLA-C*	Major histocompatibility complex, class I, C	6	8	0.75
*ACTB*	Actin beta	4	8	0.5
*CST7*	Cystatin F	4	8	0.5
*CYBA*	Cytochrome b-245 alpha chain	4	8	0.5
*FCER1G*	Fc epsilon receptor Ig	4	8	0.5
*GZMA*	Granzyme A	4	8	0.5
*IFITM2*	Interferon induced transmembrane protein 2	4	8	0.5
*IFITM3*	Interferon induced transmembrane protein 3	4	8	0.5
*LYZ*	Lysozyme	4	8	0.5
*MALAT1*	Metastasis associated lung adenocarcinoma transcript 1	4	8	0.5
*MT2A*	Metallothionein 2A	4	8	0.5
*MYOM2*	Myomesin 2	4	8	0.5
*NFKBIA*	Nuclear Factor of Kappa Light Polypeptide Gene Enhancer In B-Cells Inhibitor, Alpha	4	8	0.5
*PIM1*	Pim-1 proto-oncogene, serine/threonine kinase	4	8	0.5
*S100A8*	S100 calcium binding protein A8	4	8	0.5
*S100B*	S100 calcium binding protein B	4	8	0.5
*TSC22D3*	TSC22 domain family member 3	4	8	0.5

**Table 2:** The curated gene list with the supporting literature from PubMed. Conventional genes denote well-established NK cell or cytotoxic lymphocyte markers, whereas novel genes represent non-canonical, context-dependent regulators identified through integrative GAFA analysis in CML TFR and relapse.

Tag	Gene Symbol	PubMed IDs	Discovery
Conventional	*CST7*	29180998, 41229419	Cystatin F can be an important mediator within tumor microenvironment affecting the cytotoxicity of NK cells and consequently antitumor immune response.
	*FCER1G*	31220194, 41177540, 33239726	*FcRγ* is a signaling molecule for Fc receptors and NK cell killer activating receptor (KAR) complex. *FcRγ* is highly expressed by NK cells and involved in NK cell activity.
	*GNLY*	31107565, 20660289, 29137359	Granulysin is a well-established cytotoxic effector molecule expressed in NK cells.
	*GZMA*	19506301, 39511139	Natural killer cell-specific serine protease may function as a common component necessary for lysis of target cells by cytotoxic T lymphocytes and natural killer cells.
	*HLA-C*	32309433, 18841361	*HLA-C* is a known ligand for inhibitory KIR receptors on NK cells.
Novel	*ACTB*	38315012	A novel de novo (encoding-actin) mutation, specifically G342D, causes a functional NK cell deficiency (fNKD) by impairing the actin cytoskeleton, which results in increased cell spreading, defective lytic synapse termination, and reduced killing capacity.
	*CYBA*	30062795	Examination of lymphocyte populations—recognized as critical regulators of metastatic colonization—revealed a higher proportion of activated T cells and NK cells in the lungs of *Cyba*^tm1a^ mice compared with controls.
	*IFITM2*	40215772	*IFITM2* was associated with both sepsis-related cognitive dysfunction and Alzheimer disease.
	*IFITM3*	32553073	The NK cells are more readily activated in the absence of *IFITM3*, increasing mortality in *Ifitm3*−/− mice during acute influenza infection.
	*LYZ*	37428911	LYZ-high hepatocellular carcinomas were characteristic of the most aggressive hepatocellular carcinoma subtype, exhibiting impaired metabolic programs alongside enhanced proliferative and metastatic potential.
	*MALAT1*	28412742	MALAT1 was related to poor prognosis of NK cell lymphomas.
	*MT2A*	17622311	The +838 C/G MT2A polymorphism seems to influence inflammatory markers, zinc availability, NK cell cytotoxicity, and trace element status, all of which may promote carotid artery stenosis development.
	*MYOM2*	36405736	Unbiased clustering identified four NK cell subsets in Alzheimer disease, including a distinct expanded population marked by *MYOM2* upregulation that was negatively associated with cognitive function.
	*NFKBIA*	37607536	The CD56^dim CD16^high subset (NFKBIA) exhibited one of the highest inflammatory scores among all NK cell subsets. This cluster was characterized by predominant expression of CCL3, CCL4, and CCL4L2, suggesting an enhanced capacity to recruit additional immune cells, including T cells.
	*PIM1*	33414987	PIM1, like PIM2, is a serine/threonine kinase involved in cytokine-driven survival and metabolic regulation in immune cells. In natural killer (NK) cells, PIM1 functions downstream of IL-15/JAK–STAT signaling, where it contributes to cell survival, proliferation, and metabolic fitness.
	*S100A8*	39133305	Reduced levels of S100A8/A9, NK cells, and IFN-γ could be valuable for the treatment of liver cirrhosis with diabetes mellitus.
	*S100B*	35368338	S100B is predominantly expressed in immune cells, especially NK cell
	*TSC22D3*	22977521	The combination of high TSC22D3 expression and low GBP1 expression may be considered a risk factor for the early recurrence of hepatocellular carcinoma after liver resection.

## Data Availability

Code for the analysis above is available at https://github.com/ai-pharm-AU/CML_NK_scRNA_TKI (accessed on 3 April 2026).

## Supplementary Materials

The following supporting information can be downloaded at https://www.mdpi.com/article/10.3390/biology15070588/s1: Figure S1: Mean fraction of NK cell clusters across patient time points stratified by clinical outcome; Figure S2: Differential regulon activity along NK cell differentiation trajectories across clinical outcome samples. The heatmap represents the min–max scaled TF activities. Table S1: Biomarker expressions and associated *p*-values across cell clusters. Table S2: The performance in the eight validations. Table S3: Transcription factor list ordered by the PageRank score. Table S4: Transcription factor-associated targets and conditional up-/down-regulation. Table S5: Transcription factor-targets relationship based on SCORPION weights.

## Abbreviations

The following abbreviations are used in this manuscript:

AI	Artificial intelligence
APC-like	Antigen-presenting cell-like
AnnData	Annotated data object format
AUC	Area under the curve
BCR–ABL1	Fusion oncogene (Breakpoint cluster region–Abelson 1)
CML	Chronic myeloid leukemia
DASFREE	DASatinib treatment FREEdom; commonly referenced as DASFREE
DEG	Differentially expressed gene
DPT	Diffusion pseudotime
EURO-SKI	European Stop Kinase Inhibitor trial
FDR	False discovery rate
GAFA	Gene Regulatory Network–AI–Functional Analysis
GAM	Generalized additive model
GO	Gene Ontology
GRN	Gene regulatory network
IFN-γ	Interferon gamma
IKK	IκB kinase
IRAK1	Interleukin-1 receptor–associated kinase 1
logFC	The log 2 based fold change
MHC	Major histocompatibility complex
mTORC1	Mechanistic target of rapamycin complex 1
NF-κB	Nuclear factor kappa-light-chain-enhancer of activated B cells
NK	Natural killer (cells)
PPI	Protein–protein interaction
qPCR	Quantitative polymerase chain reaction
ROC–AUC	Receiver operating characteristic area under the curve
SCORPION	Single-Cell Oriented Reconstruction of PANDA Individually Optimized Gene Regulatory Networks
scRNA-seq	Single-cell RNA sequencing
scVI	Single-cell variational inference
STAT1/2/3	Signal transducer and activator of transcription 1/2/3
STRING	Search Tool for the Retrieval of Interacting Genes/Proteins
TF	Transcription factor(s)
TFR	Treatment-free remission
TGF-β	Transforming growth factor beta
TKI	Tyrosine kinase inhibitor(s)
TLR	Toll-like receptor
TNF-α	Tumor necrosis factor alpha
TRRUST	Transcriptional Regulatory Relationships Unraveled by Sentence-based Text mining
TCRαβ	T-cell receptor alpha–beta
UMAP	Uniform Manifold Approximation and Projection
UMI	Unique molecular identifier
