# Supplementary material for: An AI-Enabled Single-Cell Transcriptomic Analysis Pipeline for Gene Signature Discovery in Natural Killer Cells Linked to Remission Outcomes in Chronic Myeloid Leukemia
Source: Biology (Basel). 2026 Apr 6;15(7):588. doi: 10.3390/biology15070588 (PMC13072394; doi:10.3390/biology15070588)
Supplement: Supplementary file 1 [file biology-15-00588-s001.zip › CML NK TKI supplemental.pdf]

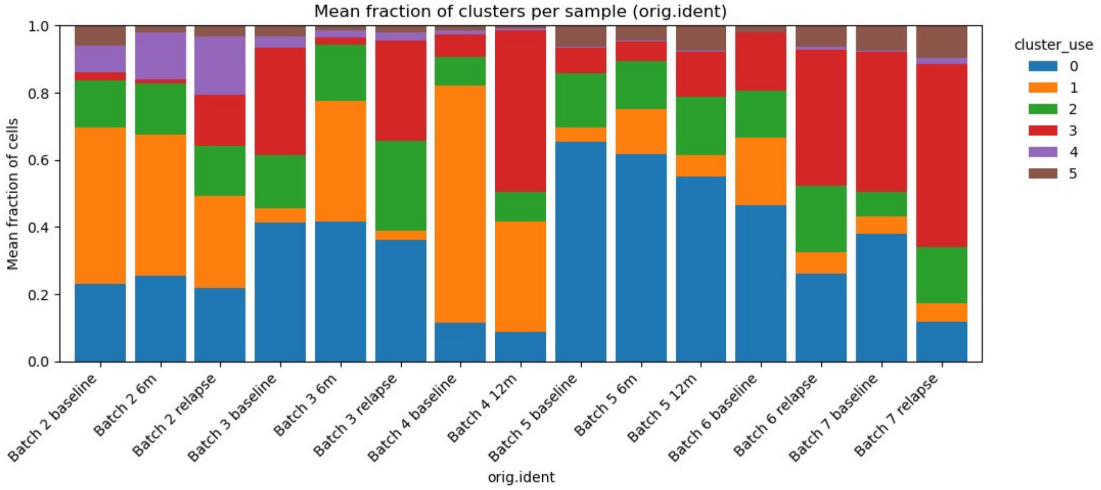

Figure S1. Mean fraction of NK cell clusters across patient time points stratified by clinical outcome. The upper bar plot shows the total number of cells per outcome group, while the lower stacked bar plot illustrates cluster composition and relative abundance across outcomes.

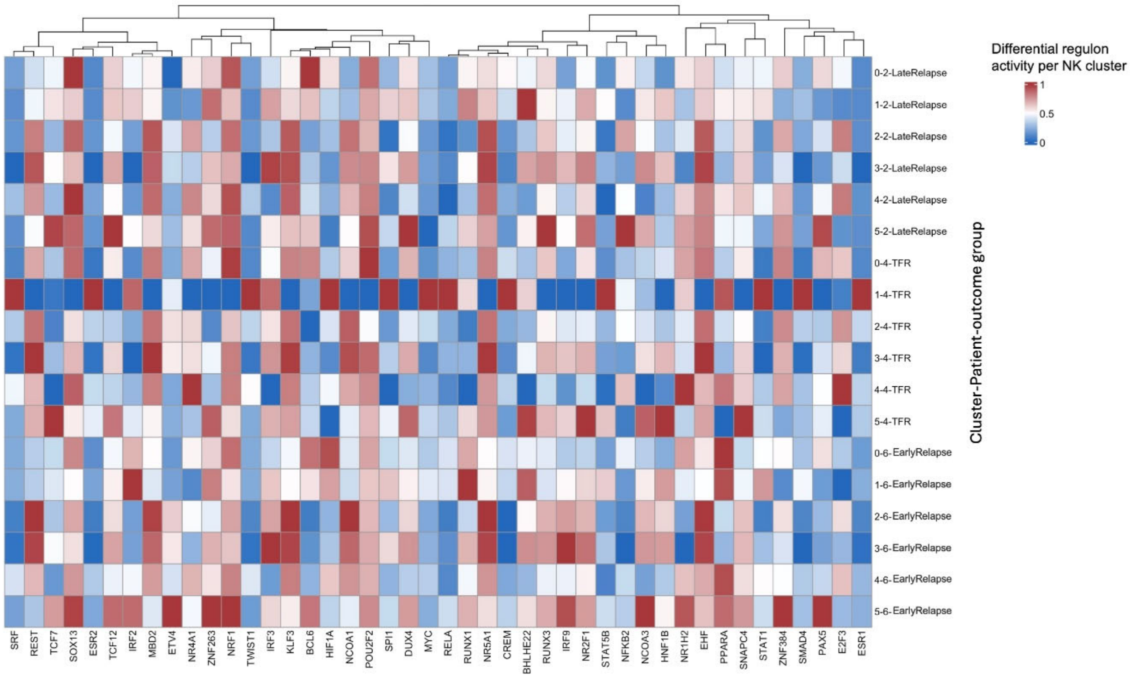

Figure S2. Differential regulon activity along NK cell differentiation trajectories across clinical outcome samples. The heatmap represents the min–max scaled TF activities.
